# Camera trapping in ecology: A new section for wildlife research

**DOI:** 10.1002/ece3.9925

**Published:** 2023-03-16

**Authors:** Jason T. Fisher

**Affiliations:** ^1^ School of Environmental Studies University of Victoria Victoria British Columbia Canada

**Keywords:** camera traps, wildlife ecology

Ecological research is undergoing a substantial transformation. Camera trapping—“capturing” a photograph remotely, allowing observation of wildlife separately from the observer—has been around for over a century. However, it emerged as a substantive mode of sampling wildlife occurrence only about three decades ago (Kucera & Barrett, 2011; O'Connell et al., 2011) and is now rapidly improving and innovating, changing the face of wildlife ecology research (Burton et al., 2015). With repeated sampling made possible across space and time, limited only by logistics and resources, wildlife observations can be gathered and analyzed at unprecedented spatial and temporal scales.

With the engineering of relatively inexpensive camera models that do not require costly support systems (such as those needed for satellite telemetry), camera traps also serve to democratize research. Camera trapping has consequently spread across the global south and developing countries (Agha et al., 2018; Cremonesi et al., 2021; Galindo‐Aguilar et al., 2022). Many private citizens run their own camera traps; networking observations from these citizen scientists have yielded great insights and will continue to do so (McShea et al., 2016). Camera traps are being employed by Indigenous peoples to ask questions about wildlife on their traditional territories (Artelle et al., 2021; Fisher et al., 2021), an important step towards meeting the principles of the United Nations Declaration on Rights of Indigenous Peoples (Gilbert, 2007).

Camera‐trap research spans the ecological hierarchy, with applications to animal behavior (Caravaggi et al., 2017, 2020) such as diel activity (Frey et al., 2017; Rowcliffe et al., 2014), populations (Bischof et al., 2020; Gardner et al., 2010), species' distributions (Rich et al., 2017; Tobler et al., 2015), and wildlife communities (Ahumada et al., 2011; Wittische et al., 2021). With adequate inferential logic and analysis, more complex ecological processes such as species interactions can also be discerned (Beirne et al., 2021; Clare et al., 2016; Niedballa et al., 2019). The field is rich for the planting seeds of new ideas. In fact, though camera trapping has largely been used for mammals, it is expanding taxonomically to include vegetation communities (Seyednasrollah et al., 2019; Sun et al., 2021), herptiles (Moore et al., 2020; Welbourne et al., 2020), and avifauna (Jachowski et al., 2015; Murphy et al., 2018).

Software has advanced in‐step with the hardware. Converting images to numerical data is made easier with custom software, much of it open‐source (Greenberg et al., 2019; Young et al., 2018). Processes for automatic identification are being developed to greatly speed up image classification and process “big data” (Duggan et al., 2021; Shepley et al., 2021). Conceptual advances, such as frameworks for understanding how camera detections sample underlying ecological processes, are paving the way for sophisticated insights (Glover‐Kapfer et al., 2019; Hofmeester et al., 2019).

Tremendous discoveries lay in the future. Networking arrays of camera traps across different landscapes—even globally, similar to weather networks (Steenweg et al., 2017)—will allow macroecological research on a scale never before possible (Chen et al., 2022; Magle et al., 2021; Rich et al., 2017). Notwithstanding, great insights await in small focal studies too—these lay the foundations of ecological inference. We support these endeavors in *Ecology & Evolution*'s new section *Camera Trapping in Ecology*. The journal's mandate to be author‐friendly, without gatekeeping assessments of importance as a barrier, makes us a place that welcomes both small‐scale autecological studies and large‐scale syntheses. This journal's philosophy is to help authors have their work read and used by the scientific community—we believe this Section will help with that goal.

*Ecology and Evolution*'s first volume featured its first camera‐trapping study (Fisher et al., 2011), a paper desk‐rejected from several other journals as being “interesting but improbable” among other traditional fare. The Editorial team gave it a chance, and over 100 citations later, it continues to stimulate scientific debate (Stuber & Fontaine, 2019). Since then, we have published 100 s of camera‐trap studies. We are eagerly anticipating many more camera‐trap papers in this dedicated Section, as *Ecology and Evolution* plans to be at the forefront of the great proliferation of camera‐trapping research, and continue to serve as a platform for scientific thought and debate.

## AUTHOR CONTRIBUTIONS

**Jason Thomas Fisher:** Conceptualization (equal); writing – original draft (equal).

## Data Availability

No data available.
